# Editorial

**Published:** 1876-02

**Authors:** 


### Editorial.

### EXAMINATIONS.

The Ohio State Board of Dental Examiners will hold a meeting for the examination of applicants under the law regulating the practice of dentistry in Ohio in the Dental College in Cincinnati, on Wednesday, March 1st, at 12 o'clock m.

All who wish to avail themselves of this opportunity, will please present themselves promptly at that time.

### LEGISLATION FOR REGULATING DENTAL PRACTICE.

The dental profession of Kentucky are moving in this matter and seem determined not to be behind their brethren of other states in their efforts to redeem dental practice from the thraldom of quackery, in which it has been so long and so deeply involved.

At a called meeting of the Kentucky Dental Society, recently held in the city of Louisville, measures were inaugurated, and we doubt not will be promptly executed, for securing a law to regulate the practice of dentistry in that state. A law was enacted about three years ago for the organization of the Kentucky State Dental Society. This gave to the dental pro fession in that state a legal recognition and status; but more than this is found necessary—some legislation that will place restraint upon quackery, and give encouragement to legitimate practice. We look with interest for the success of this effort and will be pleased to give its provisions in the pages of the REGISTER so soon as it shall have passed the legislature. We trust the cause will not be given up until the object sought is obtained.

---

### OBITUARY.

DIED, December 30th, 1875, at 10 o'clock a. m., after a long and severe illness, Dr. H. R. Smith, dentist of Cincinnati.

Dr. Smith has been for many years one of the leading practitioners of his profession in this city. He was also for many years a professor in the Ohio College of Dental Surgery.

We expect to publish in the March number of the REGISTER, a biographical sketch of the doctor and will only add to this the action of the dentists of this city in reference to his death.

"At a meeting of the dentists of this city, held at the Dental College, Dr. James Taylor presiding, the following preamble and resolutions were adopted, viz:

"Whereas, It has pleased our Heavenly Father to remove from our midst by death, our professional brother, Dr. Harris R. Smith; therefore,

Resolved. That in the death of Dr. Smith our profession sustained a loss that can hardly be estimated. He was active and efficient in whatever he engaged, and was always an ardent worker for the welfare and advancement of his profession; this he exhibited in many ways, but especially as a writer and teacher.

Resolved, That we regard it our duty to place on record an expression of our high esteem and appreciation of our departed brother, as an upright and noble man, of sterling integrity, warm-hearted and generous in all his course of life, as a friend, always reliable and true.

Resolved, That a copy of these resolutions be transmitted to the family of the deceased, with assnrance of our sincere sympathy in this, their great bereavement."

J. TAFT.

A. BERRY. } Committee.

H. A. SMITH.

### THE OHIO DENTAL COLLEGE ASSOCIATION

Will hold its annual session on Tuesday, February 29th, at 2 o'clock, at which time it is desirable that all of the stockholders of the college be present, as business of importance pertaining to the institution, will come before the meeting and the business should be promptly at the time appointed.

### COMMENCEMENT.

The Commencement Exercises of the Ohio College of Dental Surgery will be held on Thursday, March 2d, at 7½ o'clock p. m. All in any wise interested are cordially invited to be present.

The annual address upon this occasion will be delivered by Dr. W. H. Goddard, of Louisville, and the degrees conferred by the President of the Board of Trustees.

### THE DENTAL LUMINARY.

This little work prepared several years ago under the supervision of, and issued by the Cincinnati Dental Society; was gotten up with special reference to the want of the public, in reference to the care of the teeth; it is designed for distribution by dentists to their patients. It contains a number of lectures or articles upon the management and care of the teeth; upon dentition and the care of children's teeth. It also contains the Code of Ethics of the American Dental Society, and the law regulating the practice of Dentistry in Ohio. A new edition is now being issued, which can be furnished to Dentists with their card printed on the cover for from $4.00 to $7.00 per hundred, according to the style of cover, etc They can be supplied by application to the Editor of the. Register.

### IN MEMORIAM.

At a special meeting of the St. Louis Dental Society, held on Wednesday Evening, June 16th, 1875, the following resolutions of respect were unanimously adopted.

Whereas, Dr. A. W. Morrison, an active and respected member of the Society, has been removed by death from our midst, therefore

Resolved, That in the death of Dr. A. W. Morrison, the profession has lost an ardent, sincere and valuable member, and one whose many excellencies of character have endeared him to all with whom he became associated.

Resolved, That the members of the Society deeply sympathize with the relatives and friends of our departed brother, in this their sudden bereavement.

Resolved, That a copy of these resolutions be presented to the family of the deceased and that they be published in the Dental Journals. N. N. Keieh, Sect'y.

